# Supplementary material for: Design of High-Relaxivity Polyelectrolyte Nanocapsules Based on Citrate Complexes of Gadolinium(III) of Unusual Composition
Source: Int J Mol Sci. 2021 Oct 27;22(21):11590. doi: 10.3390/ijms222111590 (PMC8583736; doi:10.3390/ijms222111590)
Supplement: Supplementary file 1 [file ijms-22-11590-s001.zip › ijms-1414672-supplementary.pdf]

Electronic supplementary information

Design of high-relaxivity polyelectrolyte nanocapsules based on citrate complexes of gadolinium(III) of unusual composition

Evgenia Burilova [1,2,*], Alexander Solodov [2], Julia Shayimova [2], Julia Zhuravleva [2], Darya Shurtakova [2], Vladimir Evtjugin [2], Elena Zhiltsova [1], Lucia Zakharova [1], Ruslan Kashapov [1] and Rustem Amirov [2]

[1] Arbuzov Institute of Organic and Physical Chemistry, FRC Kazan Scientific Center, Russian Academy of Sciences, Kazan 420088, Russia; burilovajen07@mail.ru (E.B.); zhiltsova@iopc.ru (E.Z.); luciaz@mail.ru (L.Z.); rusl701@yandex.ru (R.K.);

[2] Kazan Federal University, Kremlevskaya str. 18, Kazan, Russian Federation, 420008, Russia; burilovajen07@mail.ru (E.B.); sanya.solodiv@live.com (A.S.); julia_shayimova@mail.ru (J.S.); yulialab6@mail.ru (J.Z.); darja-shurtakva@mail.ru (D.S.); rramirov58@mail.ru (R.A.); vevtugyn@gmail.com (V.E.)

* Correspondence: burilovajen07@mail.ru; Tel.: +7(843)233-71-45

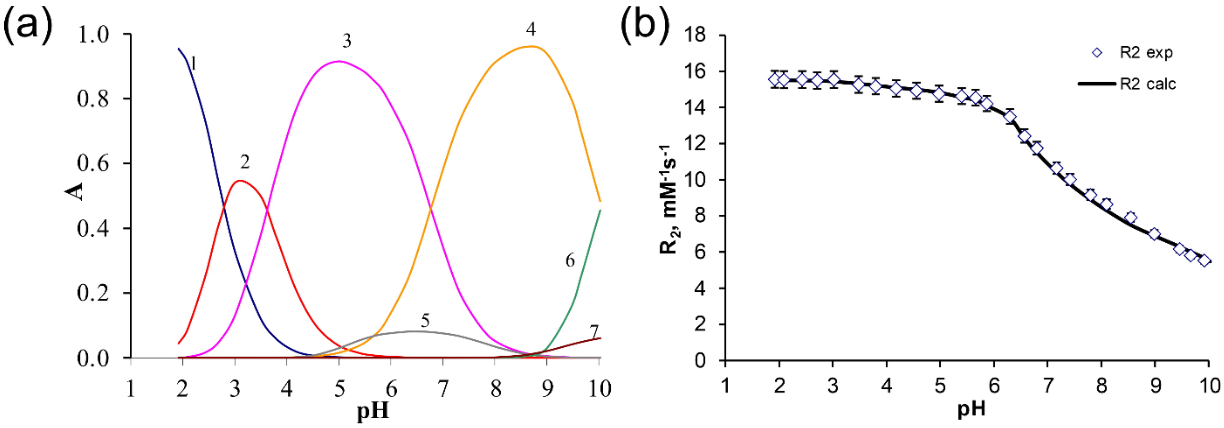

Figure S1: Diagrams of the proportional distribution of complex forms accumulating in the gadolinium(III) - citric acid system (a) and Relaxivity vs pH experimental and fitted curves (b) at a metal-ligand ratio 1 : 1 (1-Gd, 2-GdH$_2$L, 3-GdHL, 4-GdL, 5-Gd(HL)$_2$, 6-Gd$_3$L$_3$(OH)$_2$, 7-GdL$_2$)

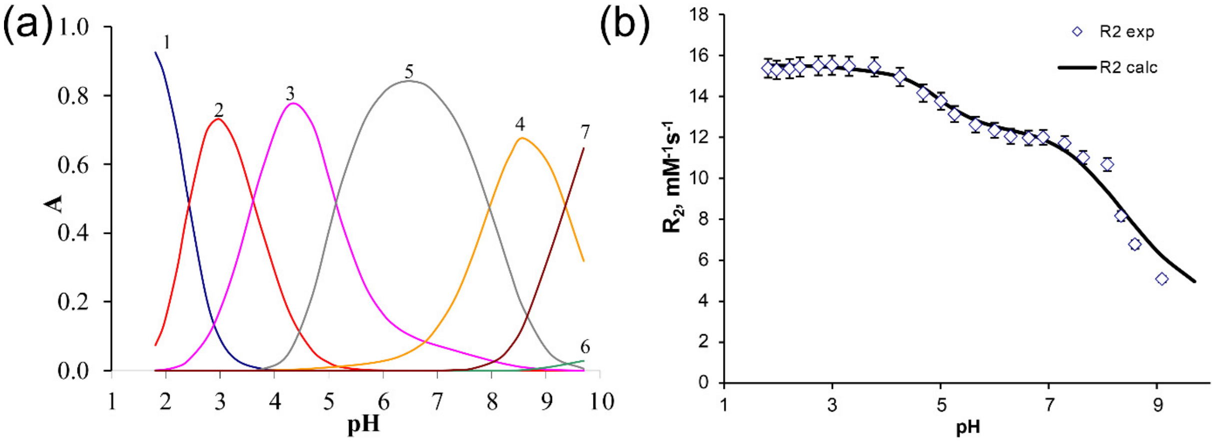

Figure S2: Diagrams of the proportional distribution of complex forms accumulating in the gadolinium(III) - citric acid system (a) and Relaxivity vs pH experimental and fitted curves (b) at a metal-ligand ratio 1 : 3; (1-Gd, 2-GdH$_2$L, 3-GdHL, 4-GdL, 5-Gd(HL)$_2$, 6-Gd$_3$L$_3$(OH)2, 7-GdL$_2$)

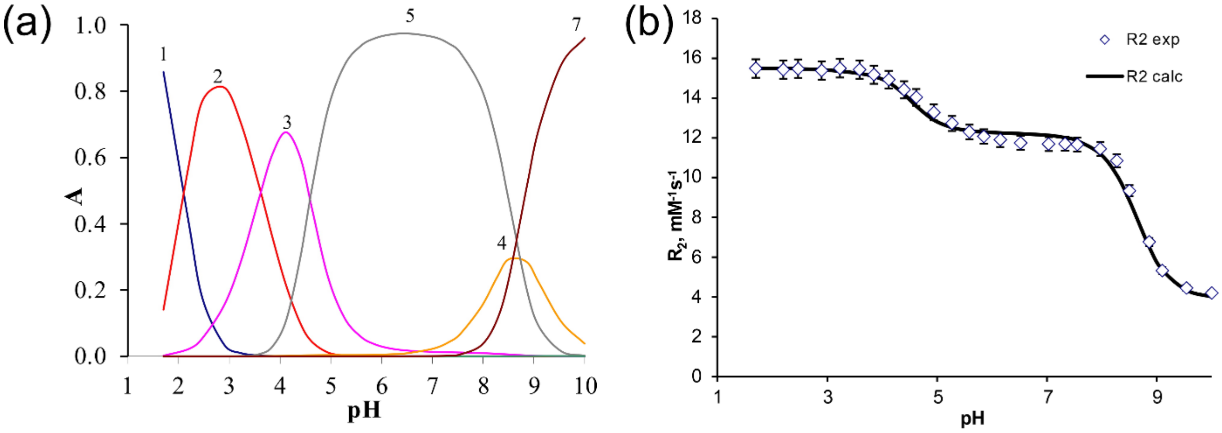

Figure S3: Diagrams of the proportional distribution of complex forms accumulating in the gadolinium(III) - citric acid system (a) and Relaxivity vs pH experimental and fitted curves (b) at a metal-ligand ratio 1 : 10; (1-Gd, 2-GdH$_2$L, 3-GdHL, 4-GdL, 5-Gd(HL)$_2$, 6-Gd$_3$L$_3$(OH)$_2$, 7-GdL$_2$)

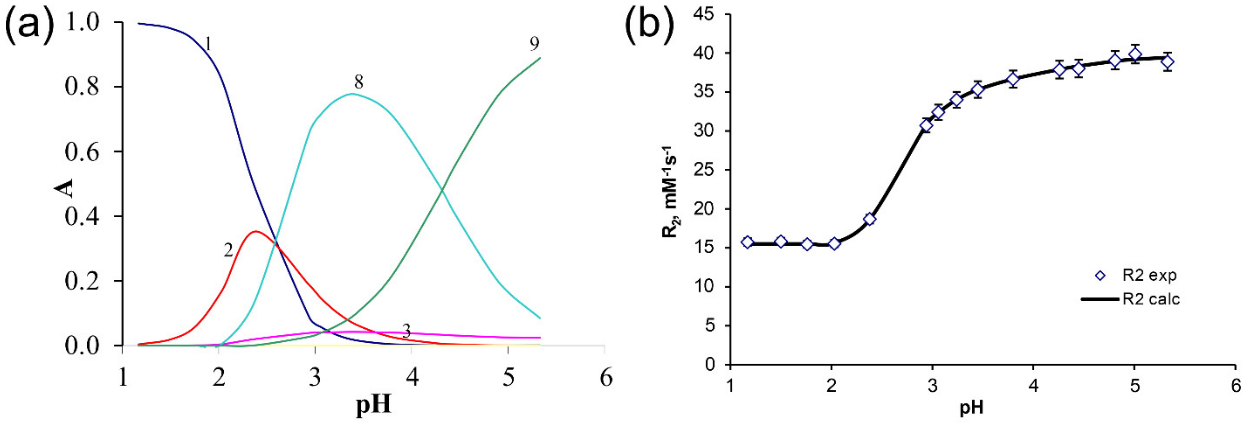

Figure S4: Diagrams of the proportional distribution of complex forms accumulating in the gadolinium(III) - citric acid - PEI0 system (a) and Relaxivity vs pH experimental and fitted curves (b) at a metal-ligand-PEI0 ratio 1 : 3 : 10. (1-Gd, 2-GdH_2L, 3-GdHL, 4-GdL, 5-Gd$(HL)_2$, 6-$Gd_3L_3(OH)_2$, 7-GdL_2, 8-Gd$(H_2L)_3$, 9-Gd$(H_2L)_2$HL)

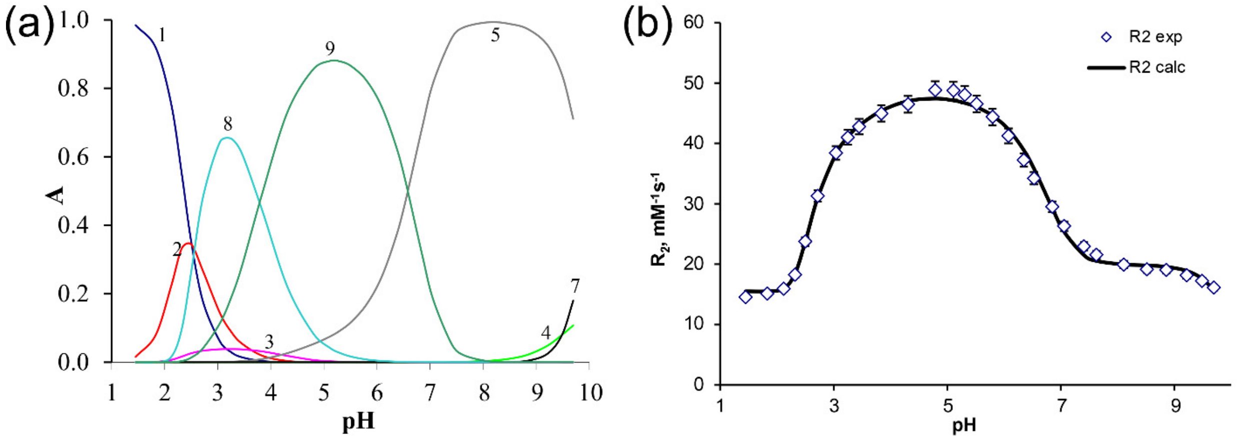

Figure S5: Diagrams of the proportional distribution of complex forms accumulating in the gadolinium(III) - citric acid - PEI0 system (a) and Relaxivity vs pH experimental and fitted curves (b) at a metal-ligand-PEI0 ratio 1 : 3 : 20 (1-Gd, 2-GdH_2L, 3-GdHL, 4-GdL, 5-Gd$(HL)_2$, 6-$Gd_3L_3(OH)_2$, 7-GdL_2, 8-Gd$(H_2L)_3$, 9-Gd$(H_2L)_2$HL)

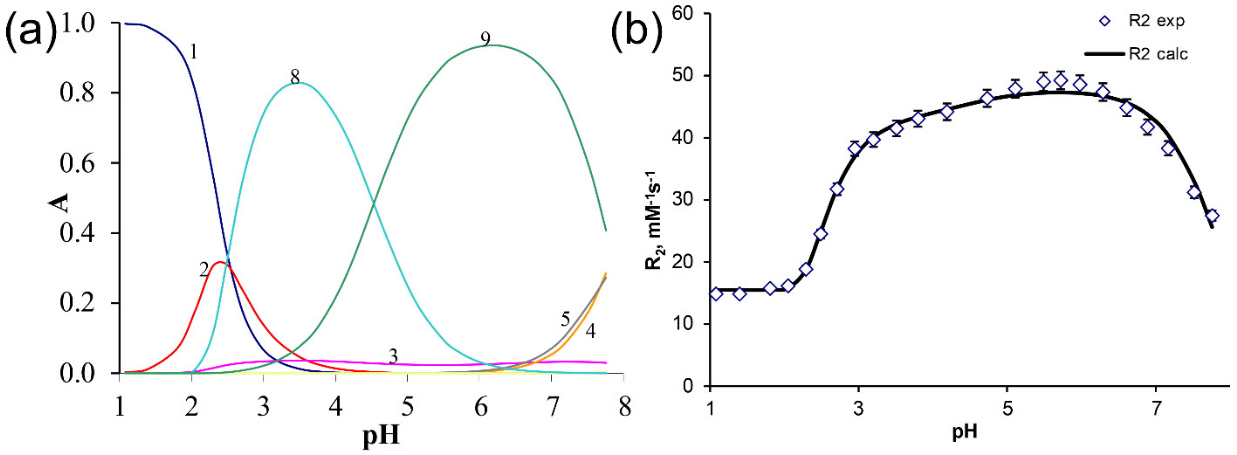

Figure S6: Diagrams of the proportional distribution of complex forms accumulating in the gadolinium(III) - citric acid - PEI0 system (a) and Relaxivity vs pH experimental and fitted curves (b) at a metal-ligand-PEI0 ratio 1 : 3 : 50 (1-Gd, 2-GdH2L, 3-GdHL, 4-GdL, 5-Gd$(HL)_2$, 6-$Gd_3L_3(OH)_2$, 7-GdL_2, 8-Gd$(H_2L)_3$, 9-Gd$(H_2L)_2$HL)

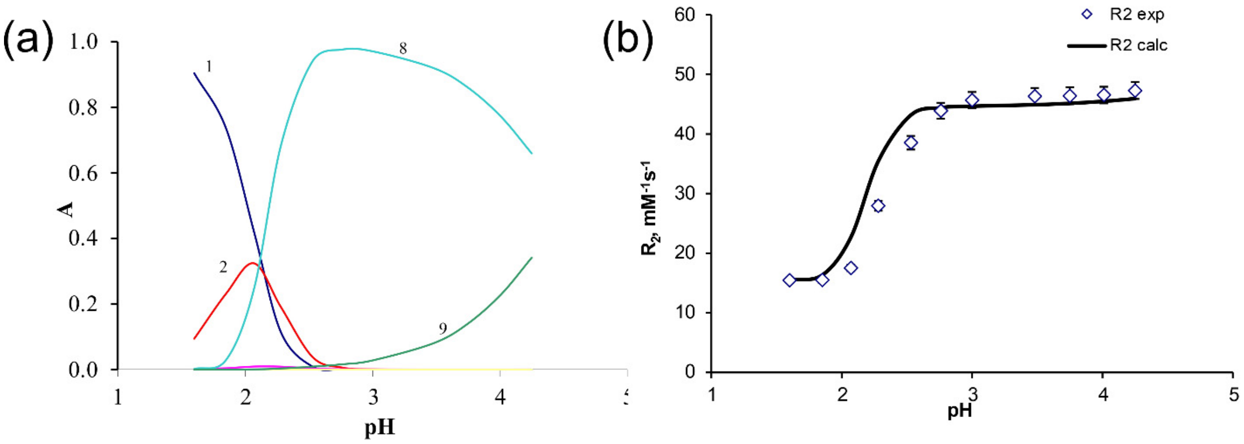

Figure S7: Diagrams of the proportional distribution of complex forms accumulating in the gadolinium(III) - citric acid - PEI0 system (a) and Relaxivity vs pH experimental and fitted curves (b) at a metal-ligand-PEI0 ratio 1 : 10 : 20; (1-Gd, 2-H_4L, 3-H_3L, 4-H_2L, 5-HL, 6-L, 7-GdH_2L, 8-GdHL, 9-GdL, 10-$Gd(HL)_2$, 11-$Gd_3L_3(OH)_2$, 12-GdL_2, 13-$Gd(H_2L)_3$, 14-$Gd(H_2L)_2HL$)

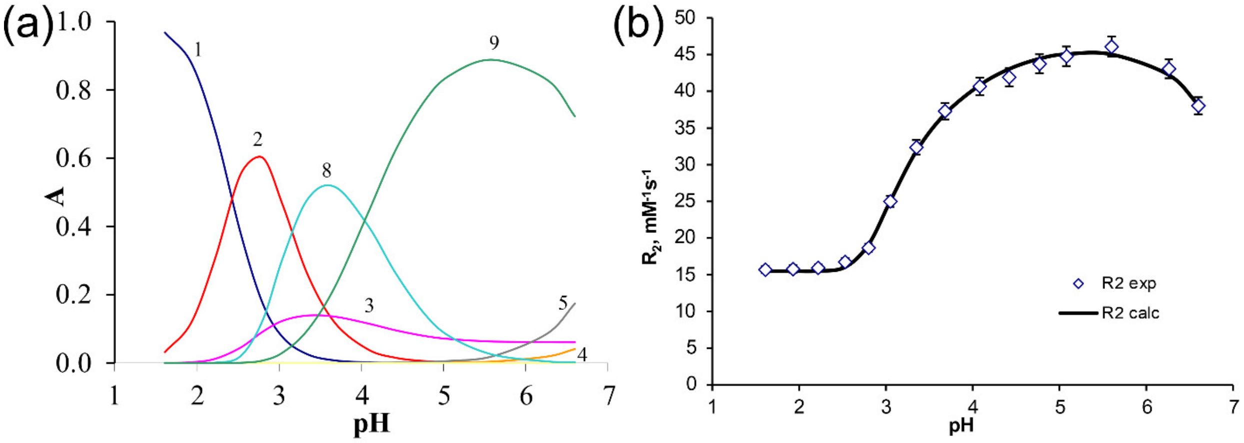

Figure S8: Diagrams of the proportional distribution of complex forms accumulating in the gadolinium(III) - citric acid - PEI0 – NaCl system (a) and Relaxivity vs pH experimental and fitted curves (b) at a metal-ligand-PEI0-NaCl ratio 1 : 3 : 20:300; (1-Gd, 2-GdH2L, 3-GdHL, 4-GdL, 5-$Gd(HL)_2$, 6-$Gd_3L_3(OH)_2$, 7-GdL_2, 8-$Gd(H_2L)_3$, 9-$Gd(H_2L)_2HL$)

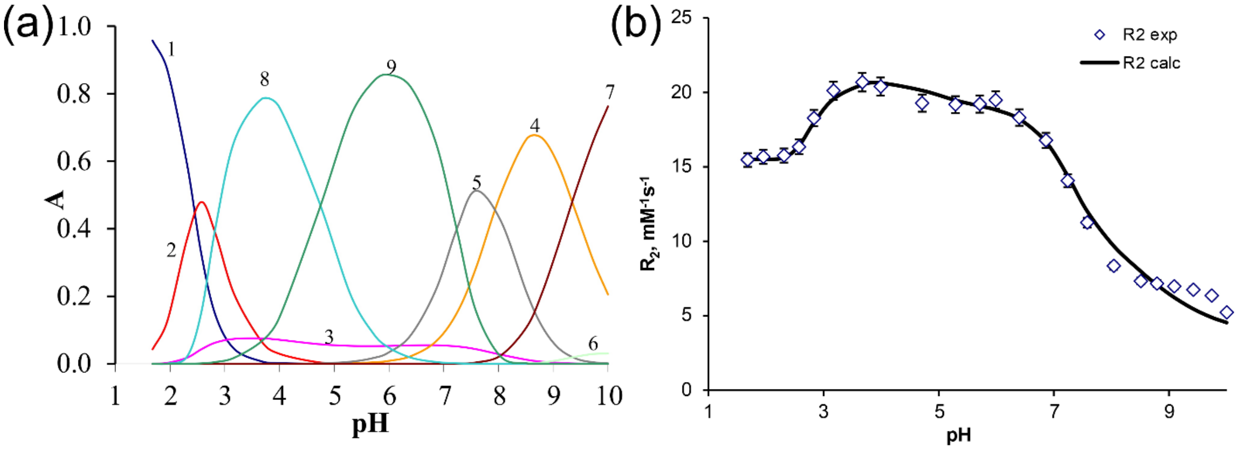

Figure S9: Diagrams of the proportional distribution of complex forms accumulating in the gadolinium(III) - citric acid – PEI3 system (a) and Relaxivity vs pH experimental and fitted curves (b) at a metal-ligand-PEI3 ratio 1 : 3 : 20; (1-Gd, 2-GdH2L, 3-GdHL, 4-GdL, 5-$Gd(HL)_2$, 6-$Gd_3L_3(OH)_2$, 7-GdL_2, 8-$Gd(H_2L)_3$, 9-$Gd(H_2L)_2HL$)

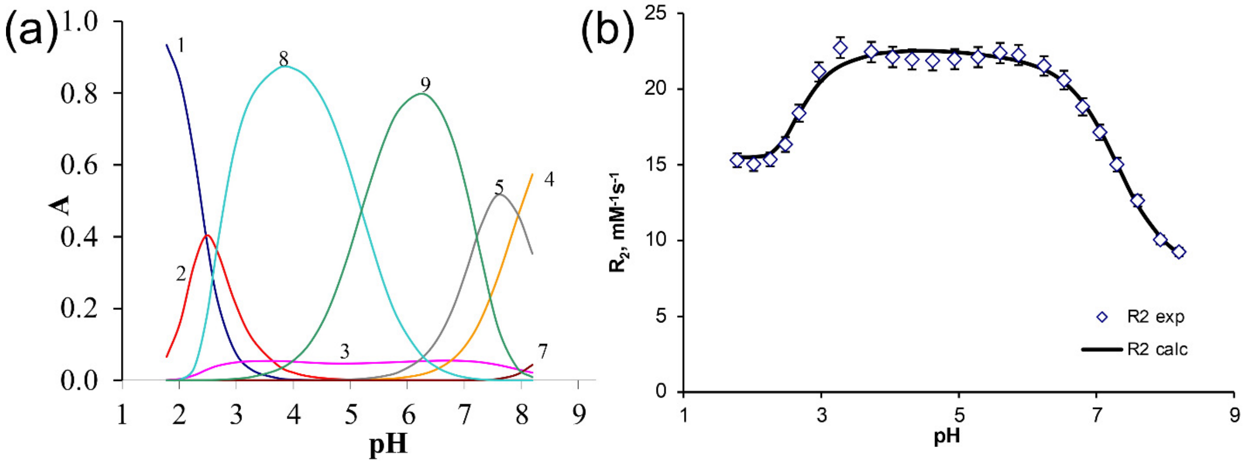

Figure S10: Diagrams of the proportional distribution of complex forms accumulating in the gadolinium(III) - citric acid – PEI2 system (a) and Relaxivity vs pH experimental and fitted curves (b) at a metal-ligand-PEI2 ratio 1 : 3 : 20; (1-Gd, 2-GdH_2L, 3-GdHL, 4-GdL, 5-$Gd(HL)_2$, 6-$Gd_3L_3(OH)_2$, 7-GdL_2, 8-$Gd(H_2L)_3$, 9-$Gd(H_2L)_2HL$)

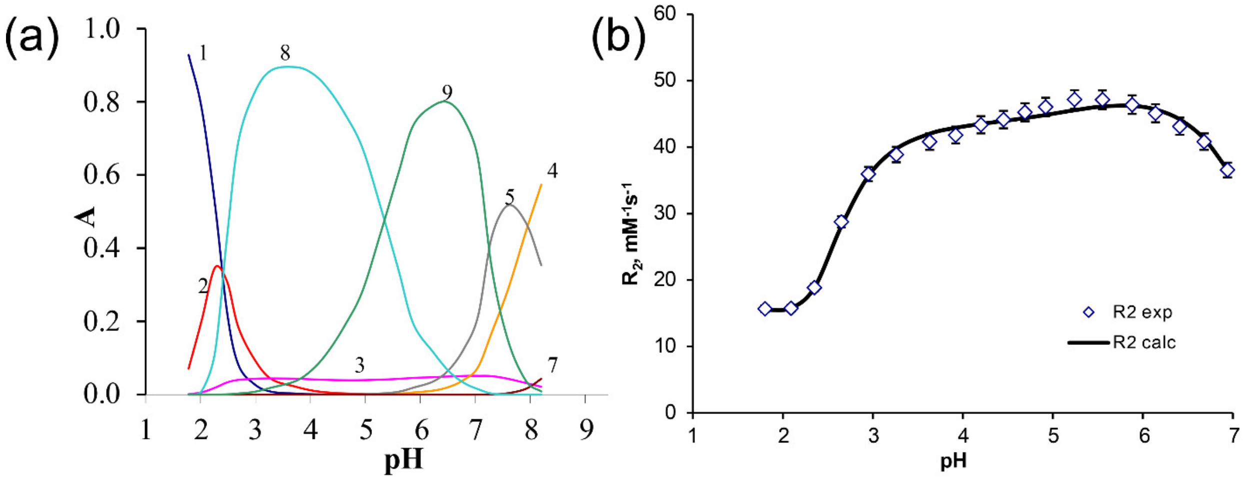

Figure S11: Diagrams of the proportional distribution of complex forms accumulating in the gadolinium(III) - citric acid – PEI1 system (a) and Relaxivity vs pH experimental and fitted curves (b) at a metal-ligand-PEI1 ratio 1 : 3 : 20; (1-Gd, 2-GdH_2L, 3-GdHL, 4-GdL, 5-$Gd(HL)_2$, 6-$Gd_3L_3(OH)_2$, 7-GdL_2, 8-$Gd(H_2L)_3$, 9-$Gd(H_2L)_2HL$)

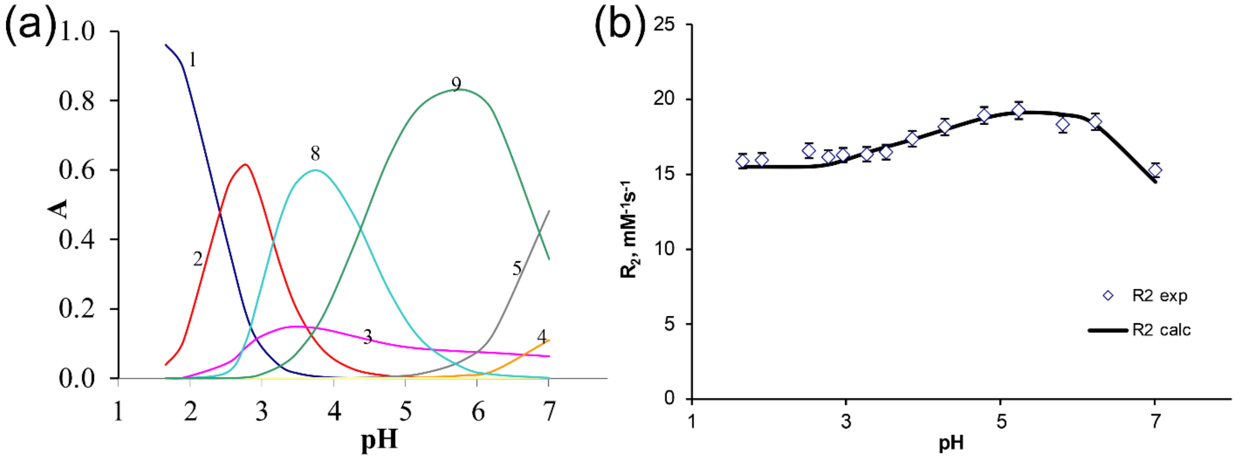

Figure S12: Diagrams of the proportional distribution of complex forms accumulating in the gadolinium(III) - citric acid – PDDC system (a) and Relaxivity vs pH experimental and fitted curves (b) at a metal-ligand-PDDC ratio 1 : 3 : 20. (1-Gd, 2-GdH_2L, 3-GdHL, 4-GdL, 5-$Gd(HL)_2$, 6-$Gd_3L_3(OH)_2$, 7-GdL_2, 8-$Gd(H_2L)_3$, 9-$Gd(H_2L)_2HL$)
